# A Virtual Escape Room versus Lecture on Infectious Disease Content: Effect on Resident Knowledge and Motivation

**DOI:** 10.5811/westjem.2021.12.54010

**Published:** 2022-01-03

**Authors:** Sara P. Dimeo, Caroline Astemborksi, Jonathan Smart, Emily L. Jones

**Affiliations:** * University of South Carolina, Greenville School of Medicine, Department of Emergency Medicine, Greenville, South Carolina; † Prisma Health Upstate, Department of Emergency Medicine, Greenville, South Carolina; ‡ University of California, Irvine School of Medicine, Department of Emergency Medicine, Orange, California; § Johns Hopkins School of Education, Masters of Education for Healthcare Professionals Program, Baltimore, Maryland

## Abstract

**Introduction:**

Medical educators are constantly seeking methods to increase engagement in the era of coronavirus disease 2019 (COVID-19) where virtual and blended learning formats are increasingly common. Educational escape rooms have previously been used to motivate learners, enhance communication skills, and cultivate teamwork. However, it is not known whether escape rooms increase learner knowledge as compared to a lecture format.

**Methods:**

This quasi-experimental study included 30 emergency medicine residents at two programs who participated in both a virtual escape room and a lecture on infectious disease content. Learners completed a pre- and post-quiz and a tool to gauge resident motivation for each activity (the Intrinsic Motivation Inventory [IMI]). The primary objective was to determine a change in knowledge as a result of the activities, and a secondary objective was to determine resident motivation for each format.

**Results:**

At both programs learners demonstrated a significant improvement in their pre- vs. post-quiz scores for the escape rooms (University of California Irvine [UCI]: 77.8% to 88.9%, p = 0.028, Prisma: 73.81% to 89.68%, p = 0.002), whereas the lectures did not impact a statistical improvement (UCI: 73.8% to 78.6%, p = 0.460, Prisma: 85.71% to 91.27%, p = 0.236). Learners at UCI noted equivalent results on the IMI for both formats, while residents at Prisma noted they were more motivated by the escape room.

**Conclusion:**

Emergency medicine residents at two programs participating in a virtual escape room demonstrated a statistical increase in knowledge on infectious disease content as compared to a lecture format and reported positive motivation ratings for both formats, with one program preferring the escape room.

## INTRODUCTION

Medical educators are constantly seeking methods to increase learner engagement, particularly in the era of coronavirus disease 2019 (COVID-19) where blended and virtual learning formats are increasingly common. One innovative modality of teaching used by educators is escape rooms. As described by Nicholson,^1^ escape rooms are defined as “live-action team-based games where players discover clues, solve puzzles, and accomplish tasks in one or more rooms in order to accomplish a specific goal (usually escaping from the room) in a limited amount of time”. Over the past five years, escape rooms have been implemented in medical education for various purposes, including recruitment to nursing programs,^2^ promoting active learning and engagement,^3^,^4^,^5^ developing teamwork and communication skills,^6^ teaching specific skills or knowledge,^7^,^8^,^9^ and fostering interprofessional development.^10^,^11^,^12^ In a systematic review by Veldkamp et al.,^13^ the vast majority of studies reported the escape room created an active learning environment with engaged learners and were highly rated by participants. Virtual escape rooms are adaptations of in-person escape rooms where the content is delivered synchronously online using tools such as Zoom (Zoom Video Communications, San Jose, CA) breakout rooms and Google Forms (Google, LLC, Mountain View, CA) to allow learners to solve a series of puzzles.

Few experimental studies have shown an increase in knowledge in a pre- vs post-test fashion,^5^,^14^,^15^ and others have demonstrated either no change^16^ or a decline in pre- to post-test scores.^17^ None of the studies had a control group. There are also no known studies to date comparing lecture formats to escape rooms. Didactic lectures remain the primary means of disseminating information to learners, with many educators using synchronous tools such as Zoom to deliver content due to the COVID-19 pandemic.^18^ Recent research has indicated that residents may be less engaged and distracted by non-conference activities during synchronous virtual didactic learning.^19^

**Figure 1:**
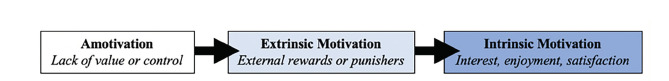
Self-determination theory continuum model.

Escape rooms have a theoretical basis to motivate learners as described by self-determination theory. The theory states that motivation comprises three psychological needs: competence; autonomy; and social relatedness. As stated by Guckian et al, 20 “a good escape room…sets achievable goals for participants (competence), facilitates freedom of choice for learners (autonomy) and features effective teamwork and facilitation (relatedness).” Self-determination theory (Figure 1) describes a continuum of motivation from a complete lack of motivation to extrinsic motivation (the provision of external rewards such as a prize or penalty) to the ultimate goal, which is intrinsic motivation or internal interest, enjoyment, and satisfaction from completion of the activity.^21^ Ideally, an escape room will fulfill learners’ needs as described by self-motivation theory.

As learning environments transform, innovative modalities of teaching that motivate learners must be urgently explored and researched in an experimental fashion. Therefore, in this study we sought to understand whether a virtual escape room on infectious disease topics would increase learner knowledge as compared to a didactic lecture, as assessed by a pre- and post-quiz. A secondary objective was to assess learners’ self-rated interest and enjoyment with the activities as determined by the Intrinsic Motivation Inventory (IMI) held after the escape room.

**Population Health Research Capsule**

What do we already know about this issue?

*Educational escape rooms have previously been shown to motivate learners, however it is not known if they positively impact knowledge compared to a lecture format*.

What was the research question?

*Does a virtual escape room on infectious disease topics increase knowledge compared to a lecture format?*

What was the major finding of the study?

*Learners improved their pre vs. post-quiz scores on escape room content, but not the lecture content*.

How does this improve population health?

*A virtual educational escape room may be a unique method to engage learners in an online synchronous format without sacrificing knowledge acquisition*.

## METHODS

We surveyed 30 emergency medicine (EM) resident learners at two different postgraduate year (PGY) 1–3 EM programs, the University of California-Irvine (UCI) in Orange, California, and Prisma Health-Upstate in Greenville, South Carolina, in March 2021. Residents at both programs were selected by convenience sample as attendees at a weekly didactic conference during their infectious disease block in March–April 2021. Study participation was voluntary. This study was determined to be exempt after review by the Prisma Health-Upstate Institutional Review Board.

Prior to study implementation, three faculty with fellowships in medical education developed six learning objectives related to infectious disease topics guided by the Model of the Clinical Practice of Emergency Medicine Practice^22^ as the basis for the lectures and escape rooms. These objectives were divided into objectives 1–3 (opportunistic infections, vector-borne illnesses, and sexually transmitted infections) and objectives 4–6 (infectious rashes, foodborne illnesses, and infectious causes of neuromuscular blockade). We crosschecked to ensure that the objectives were maintained at the same level on Bloom’s taxonomy (“remember” and “understand”). We then created and reviewed multiple-choice questions relevant to the objectives comprising the pre- and post-quizzes. The entire activity took approximately 30 hours to create, and there was no associated cost.

**Figure 2:**
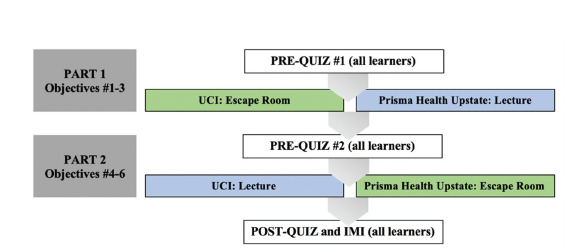
Research study design. *UCI*, University of California, Irvine; *IMI*, Intrinsic Motivation Inventory

On the study dates, participating residents completed a survey that included basic demographics, including their PGY year, identified gender, identified generation based on birth year, and previous experience with educational escape rooms. Then they completed a nine-item, multiple-choice pre-quiz relevant to objectives 1–3. After the pre-quiz, residents from UCI participated in the escape room format, whereas residents from Prisma received the same content in a lecture format. During the second half of the session, both programs completed another nine-item, multiple-choice pre-quiz, this time covering objectives 4–6. Residents from UCI this time received the lecture format, whereas residents from Prisma instead participated in an escape room activity (Figure 2). Directly after completion of all activities, learners were given the same quiz content as the pre-quizzes presented as an 18-question, multiple-choice post-quiz that covered all objectives 1–6. They also completed items from the IMI, a validated tool containing an interest/enjoyment subscale that is considered effective to assess learners’ self-reported intrinsic motivation based on self-determination theory and has been found to be adaptable to multiple research settings.^23^,^24^ (See Appendix B.)

The entire session lasted 90 minutes total, including 25 minutes for each activity followed by 5–10 minutes for debrief and questions. All content was delivered virtually via Zoom. The lecture format was delivered using Google Slides. To maintain the highest quality of lecture we used best practices in multimedia design based on Mayer’s principles of multimedia learning,^25^ including limiting the amount of text on slides and using non-distracting and enhancing graphics, as well as using color and bolding to highlight key information. A video detailing the instructions for the escape room, logistics of play, and the game rules was delivered prior to the activity.

The residents were randomly divided into teams of 4–5 participants that included a mix of PGY levels. They were split into breakout rooms and provided with a quick response code linking to a Google Form, which guided them through four escape room puzzles with a 25-minute time limit. They were allowed to use any source for information and up to two hints provided at the study authors’ discretion. The first team that completed all the puzzles correctly was recognized as the winner. See Appendix A for puzzle examples. A short debrief was held after the activity to review the escape room answers. Time was recorded by a timekeeper to ensure equal time was provided to the lecture and escape room activity.

Survey data was stored in a secure Research Electronic Data Capture survey tool (REDCap, Vanderbilt University, Nashville, TN). We calculated mean aggregated scores on the pre- and post-quizzes for the escape room and the lectures using the two proportions z-test, as well as IMI results for each program using the paired t-test for normally distributed data and the Wilcoxon signed-rank test paired for non-normally distributed data (where the Shapiro Wilks test was used to determine normality). Data analysis was conducted using the software program R version 4.0.4 (The R Foundation for Statistical Computing, Vienna, Austria).

## RESULTS

A total of 30 EM residents participated in this quasi-experimental study, 14 from UCI and 16 from Prisma. The demographics were similar at both programs, with >90% of the residents self-identifying as being born in the millennial generation (see Table 1). There were more self-identified male than female participants at both programs, with three females and 11 males from UCI, whereas there were five female and 11 males from Prisma. There was good representation across all PGY levels at both programs, with all residents being PGY1-PGY3. Most residents had only participated in 1–2 escape rooms for educational purposes in the past, with some residents at UCI having participated in more than five educational escape rooms.

**Figure 3:**
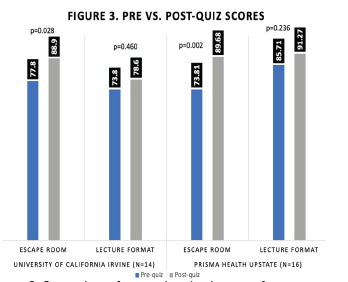
Comparison of pre- and post-quiz scores for escape room and lecture format by institution.

Residents at both programs improved their pre- vs post-quiz scores on the content related to the escape room; however, there was no significant improvement in the pre- vs post-quiz scores pertaining to the lecture activities at either program (see Figure 3).

The IMI interest/enjoyment subscale results are listed in Table 2. Learners at UCI responded to the question “I enjoyed the activity very much” with a median score of 5 for both the escape room and lecture formats (*p* = 0.1434), whereas Prisma reported a significant difference in the median score of 6 for the escape room format vs 3.5 for the lecture format (*p* = 0.0145). Learners at UCI responded to the question “The activity did not hold my attention at all” with a median score of 2 for both the escape and lecture formats (*p* = 0.4606), whereas learners at Prisma reported a significant difference of 0 for the escape room format vs 3 for the lecture format (*p* = 0.0259).

## DISCUSSION

This study demonstrated a statistically significant increase in knowledge as a result of participation in an escape room at two EM residency programs compared to a lecture format, where there was no statistical increase in knowledge. The IMI results demonstrate that residents enjoyed the escape room and found it interesting at both programs, although the learners at Prisma noted a statistical difference in their enjoyment vs the learners at UCI. While no qualitative data was collected to learn why this distinction existed, residents at UCI reported slightly more experience with educational escape rooms. Also, in our experience UCI includes more educational games as part of its didactics as compared to Prisma. A novelty effect, or a waning motivation level, has been described in previous gamification literature and could explain this difference.^26^ Despite these results, based on our observations, the use of multimedia and interactive puzzles to solve a challenge did seem to engage the learners at both programs.

## LIMITATIONS

There are many potential limitations of this study. Firstly, it was a limited convenience sample of residents attending their weekly didactic conference at two programs. There may be baseline differences in the participants that were not identified, such as their enjoyment of gamification techniques, an inherent variation in resident baseline knowledge on infectious disease topics due to different curricula at different programs, and varying familiarity with escape rooms, and in particular virtual escape rooms. The pre- and post-quizzes were the same for a given activity; therefore, recall of the questions may have affected the results (although there was still only a significant increase in the escape room groups). Competition and the increased cognitive load of the game itself could have negatively affected some learners.

It is not clear whether learners will retain the knowledge they gained through the escape room as opposed to a lecture format as we did not assess for this. While every attempt was made to ensure consistency across the content delivered, there is a possibility that the content was not presented in a similar manner, as it was delivered on two different days and the learning objectives were delivered in opposite formats to the program. The faculty did train together prior to the sessions to rehearse the teaching scripts and used similar templates for both the escape room and lecture content despite it covering different objectives. Regarding the IMI results, despite it being a validated tool this was a limited sample size and therefore may not have accurately reflected learners’ motivation.

## CONCLUSION

As a result of the COVID-19 pandemic, it has become apparent that educators must be able to adapt to virtual settings to reach their learners. Delivering a virtual escape room may be a feasible way to do this. This study helps establish the utility of using escape rooms to enhance learning as compared to a lecture format. While didactic lectures remain an efficient way for medical educators to disseminate information to learners, virtual escape rooms may be an equally if not a more effective way to provide knowledge to learners while creating a fun, motivating, and interactive environment for learning with minimal to no cost. Future research comparing traditional teaching methods to in-person escape rooms may be helpful, as well as testing long-term retention of knowledge as a result of the activities.

## Supplementary Information



## Figures and Tables

**Table 1:** Participant demographic information: University of California Irvine and Prisma Health-Upstate.

Question	UCI (n=14)	Prisma (n=16)
Generation, n (%)		
Gen X	1 (7.14)	0 (0)
Millennial	13 (92.9)	15 (93.8)
Gen Z	0 (0)	1 (6.3)
Gender, n (%)		
Female	3 (21.4)	5 (31.3)
Male	11 (78.6)	11 (68.8)
PGY Year, n (%)		
PGY1	4 (28.6)	8 (50)
PGY2	5 (35.7)	3 (18.8)
PGY3	5 (35.7)	5 (31.3)
Experience, n (%)		
I have never participated in an escape room for educational purposes	4 (28.6)	4 (25)
I have participated in a few (1–2) escape rooms for educational purposes	5 (35.7)	9 (56.3)
I have participated in multiple (3–4) escape rooms for educational purposes	3 (21.4)	3 (18.8)
I have participated in a lot of (5+) escape rooms for educational purposes	2 (14.3)	0 (0)

*Gen X*, Generation X (birth years mid-1960s to early 1980s); *Gen Z*, Generation Z (birth years mid-1990s to early 2010s; *PGY*, postgraduate year.

**Table 2:** Intrinsic motivation inventory interest/enjoyment subscale results by program.

IMI Item	UCI (n = 14)			Prisma (n = 16)		

	Escape Room	Lecture	*p*-value	Escape Room	Lecture	*p*-value
I enjoyed the activity very much. (Mean ± SD)	5.18 ± 1.17	4.44 ± 1.01	0.12	5.50 ± 1.41	4.00 ± 2.13	0.00
Median (IQR)	5 (5, 5.5)	5 (4, 5)	0.14	6 (5, 6.25)	3.5 (2, 6)	0.01
The activity was fun to do. (Mean ± SD)	4.69 ± 1.49	3.50 ± 1.09	0.03	5.44 ± 1.55	3.38 ± 2.29	0.00
Median (IQR)	5 (4, 5)	4 (2.75, 4)	0.02	6 (5, 6.25)	3 (1, 5)	0.01
I would describe the activity as very interesting (Mean ± SD)	4.50 ± 1.78	3.80 ± 1.14	0.28	5.13 ± 1.85	3.58 ± 2.19	0.00
Median (IQR)	4 (3.75, 5.5)	4 (3.25, 4.75)	0.29	6 (4, 6.5)	3.5 (2, 5)	0.01
I thought the activity was quite enjoyable. (Mean ± SD)	4.43 ± 1.83	4.25 ± 0.71	0.87	5.56 ± 1.71	3.31 ± 2.10	0.00
Median (IQR)	5 (3.25, 5)	4 (4, 5)	0.92	6 (5, 7)	3 (2, 5)	0.01
While I was doing the activity, I was thinking about how much I enjoyed it. (Mean ± SD)	3.00 ± 2.04	3.29 ± 1.25	0.84	4.67 ± 1.88	2.23 ± 2.65	0.00
Median (IQR)	3.5 (1, 4)	4 (2, 4)	1.00	5 (4, 5.5)	1 (0, 4)	0.01
I thought the activity was boring. (Mean ± SD)	2.00 ± 1.60	3.15 ± 1.14	0.12	1.06 ± 1.06	3.08 ± 2.43	0.01
Median (IQR)	2 (0.75, 3.25)	3 (2, 4)	0.16	1 (0, 2)	3 (1, 5)	0.02
The activity did not hold my attention at all. (Mean ± SD)	2.00 ± 1.71	2.70 ± 1.64	0.37	0.81 ± 1.22	3.23 ± 2.45	0.02
Median (IQR)	2 (0.75, 3.25)	2 (2, 3)	0.46	0 (0, 1)	3 (1, 5)	0.03

*UCI*, University of California, Irvine; *IMI*, Intrinsic Motivation Inventory; *SD*, standard deviation; *IQR*, interquartile range.
