# Supplementary material for: A Virtual Escape Room versus Lecture on Infectious Disease Content: Effect on Resident Knowledge and Motivation
Source: West J Emerg Med. 2022 Jan 3;23(1):9–14. doi: 10.5811/westjem.2021.12.54010 (PMC8782142; doi:10.5811/westjem.2021.12.54010)
Supplement: Supplementary file 1 [file wjem-23-9-s001.docx]

**APPENDICES**

***Appendix A: Escape Room Stations and Puzzles***

Several common puzzle designs utilized in commercial escape rooms were implemented for this educational escape room. These specific puzzles were chosen due to their ease in use in a digital environment using graphic design elements. The four chief puzzles utilized were a rebus puzzle, an acrostic cipher, a modified book cipher, and a pigpen cipher (also known as a freemason cipher). These puzzles were arranged in relative order of ascending difficulty in the linear progression of the game. Further descriptions and examples may be seen below.

1. Rebus Puzzle – A visual puzzle in which words are represented by a series of pictures and letters which must be connected phonetically to discover a secret message. In this puzzle seen in Figure 1 the correct answer is “Ivermectin” which when input would unlock the next section of the Google Form.

*Figure 1: Rebus Puzzle*


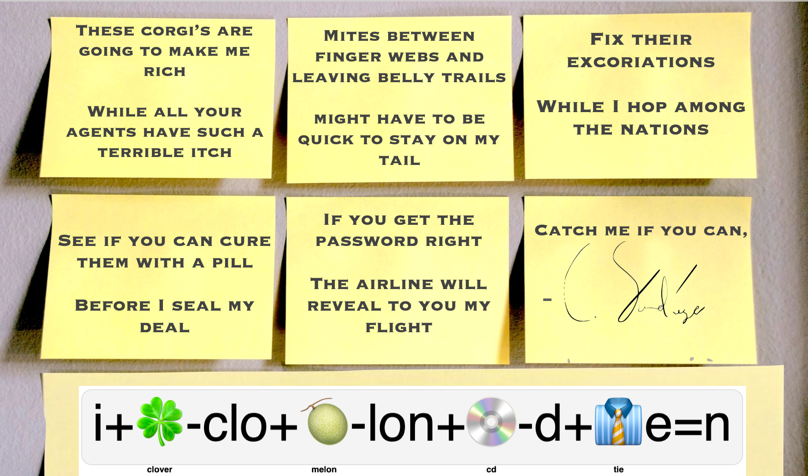


1. Acrostic Cipher – A puzzle in which the first letter of each word spells out a secret message when placed in the correct order. In this case players were presented with two slides, Figure 2 as seen with a series of masks accompanied by descriptions of common sexually transmitted infections, and Figure 3 with a series of playing cards overlaid with potential treatments. Players were required to match the correct treatment for each sexually transmitted infection. The order of the playing cards then enabled players to sort the bolded letters on the mask puzzle in ascending order to spell “Carmen” which unlocked the next Google form segment.

*Figure 2: Acrostic Cipher Puzzle Mask Stimulus*

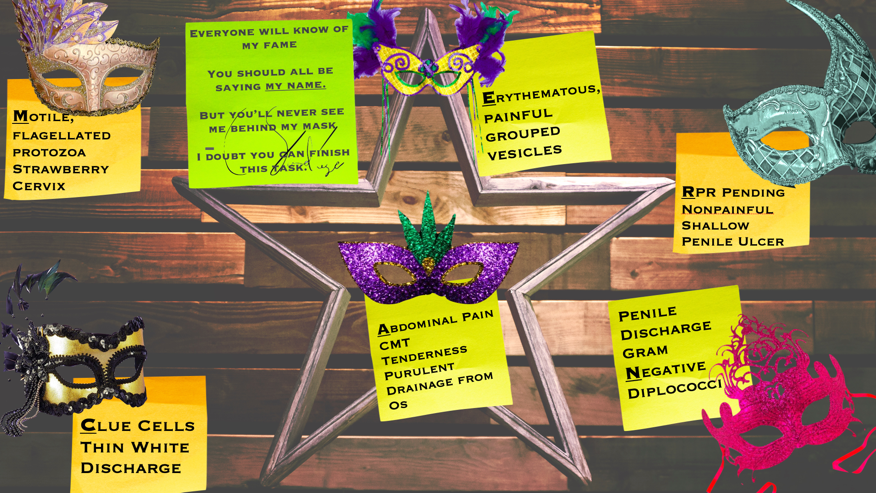


Figure 3: Acrostic Cipher Puzzle Card Stimulus


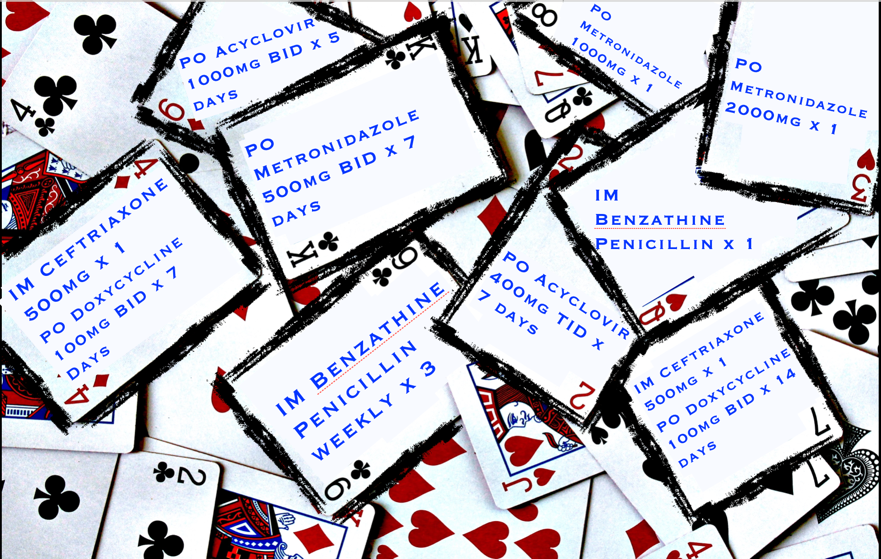


1. Modified Book Cipher – A book cipher commonly uses a book or other text as a key by which to interpret a numerical code into a series of letters to spell a message. In the modified version used for this escape room, players needed to determine which text description, as seen in Figure 4, would be best matched which chest xray visual stimulus. An example of one of the xray stimuli is seen in Figure 5. When these letters were sorted correctly the name of Carmen’s theoretical henchman “ChadPu” was given and unlocked the next Google Form section.

*Figure 4: Modified Book Text Stimulus*
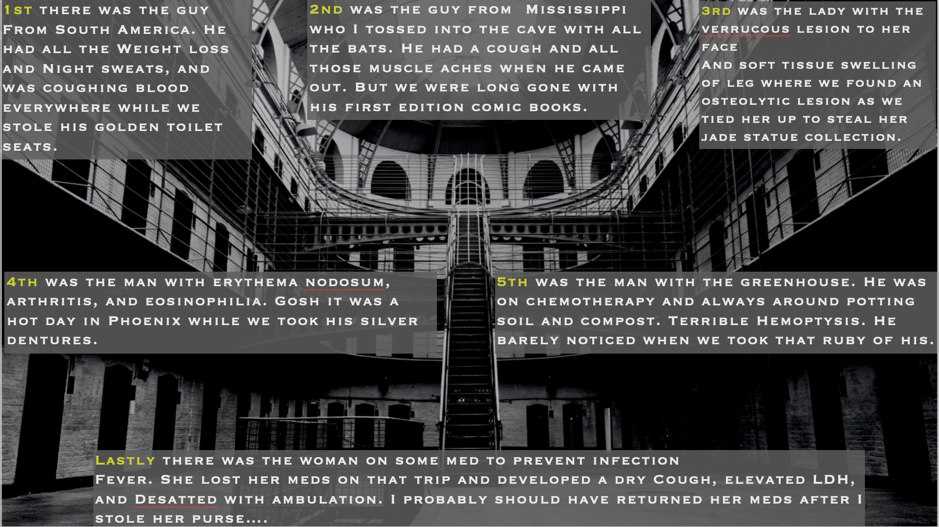

*Figure 5: Modified Book Example Photo Stimulus*


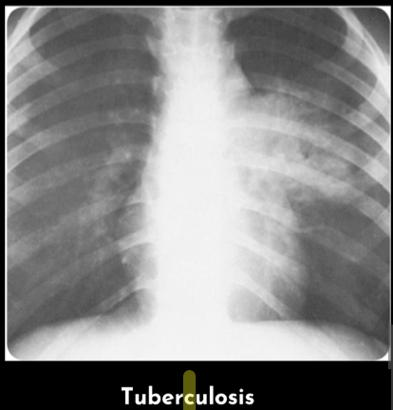


1. Pigpen Cipher (Freemason) – A pigpen cipher is a geometric substitution cipher in which the shape or symbol surrounding a letter is used to symbolize that letter in a coded message. In this case the key to the pigpen cipher is seen in Figure 6. The coded letters “inscribed” upon bottles seen in Figure 8 resulted in the partial name of a common gastrointestinal infection when translated using the cipher key. When the clue associated with the infection as seen in Figure 7 was correctly interpreted, the accompanying numbers (from left-to-right already sorted in “rainbow order”) would yield the code “2107943” which would unlock the final segment of the Google Form.

*Figure 6: Pigpen Cipher Key*


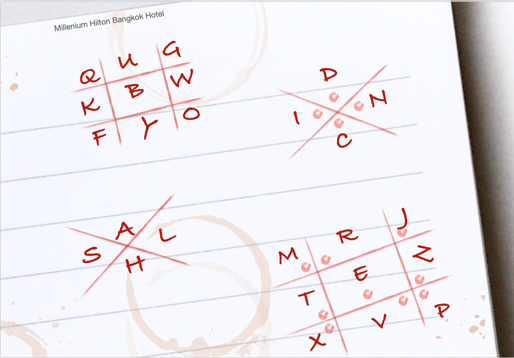

*Figure 7: Pigpen Puzzle Clues*


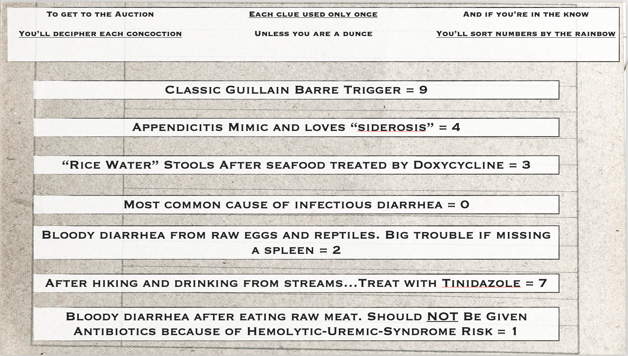

*Figure 8: Pigpen Bottle Puzzle*


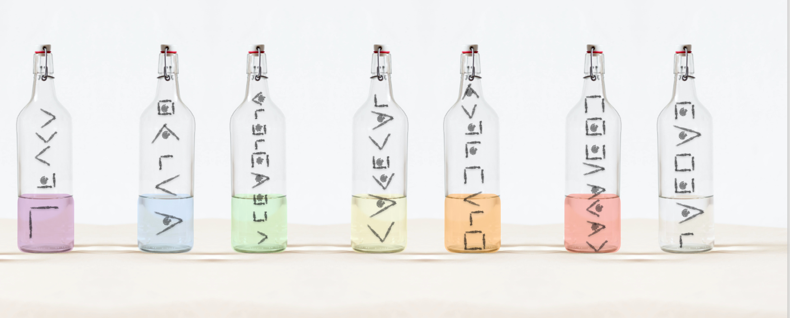


***Appendix B: REDCap Surveys***

**Demographics Survey**

1. What Generation were you born in?
2. Baby Boomer (Years 1946-1964)
3. Gen X (Years 1965-1980)
4. Millennial/GenY (Years 1981-1996)
5. Gen Z (Years 1997 – 2012)
6. What is your gender?
7. Female
8. Male
9. Non-Binary
10. Prefer not to answer
11. What is your post graduate year (PGY)?
12. PGY1
13. PGY2
14. PGY3
15. How many escape rooms have you participated in for educational purposes?
16. 0, I have never participated in an escape room for educational purposes
17. 1-2, I have participated in a few escape rooms for educational purposes
18. 3-4, I have participated in multiple escape rooms for educational purposes
19. 5+, I have participated in a lot of escape rooms for educational purposes

**Cumulative Post-Quiz: Learning Objectives 1-3** (opportunistic infections, vector-borne illnesses, and sexually transmitted infections)

1. A 54-yo alcoholic male living in rural Arizona presents with shortness of breath, fever, skin ulcers and weight loss. An initial chest x-ray shows a cavitary lesion with an air fluid level in the right lung. Multiple sputum samples are negative for acid fast bacilli.

Which pathogen is most likely the cause of these findings?

1. ***Coccidioides immitis***
2. Pneumocystis jirovecil
3. Sporothrix schenckii
4. Mycobacterium Tuberculosis
5. A 40-year-old healthy man from Ohio presents to the emergency department for 1 week of fever, cough, and myalgias. He reports camping in remote caves 2 weeks ago. Examination is significant for a temperature of 38°C, clear lung fields, normal mental status examination, and no distress. Chest X-ray reveals hilar adenopathy. Which of the following is the most likely diagnosis?
6. Blastomyces dermatitidis
7. Coccidioides immitis
8. ***Histoplasma capsulatum***
9. Pneumocystis jirovecii
10. A 48-year-old man presents to the ED complaining of painful vision loss in his left eye. He is on a four-drug treatment regimen for pulmonary tuberculosis. Which of the following medications is most likely responsible for his visual disturbance?
11. ***Ethambutol***
12. Isoniazid
13. Pyridoxine
14. Zidovudine
15. 18-year-old man presents with penile discharge after unprotected sex. He reports multiple sexual partners. Which of the following is an appropriate treatment regimen?
16. Ceftriaxone 125 mg IM x 1 and azithromycin 1,000 mg PO x 1
17. ***Ceftriaxone 500 mg IM x 1 and doxycycline 100 mg PO BID x 7 days***
18. Ciprofloxacin 500 mg BID x 7 days and azithromycin 1,000 mg PO x 1
19. Wait for the lab result in order to treat specifically
20. A 19-year-old man, who is a college student, presents to the ED with concern for a lesion on his penis for the past two days. He began a relationship with a new sexual partner three weeks ago. On exam, there is a nontender 1 cm ulcer on the dorsum of his glans. There is no inguinal adenopathy.  What is the most appropriate treatment?
21. Azithromycin
22. Ceftriaxone
23. Ciprofloxacin
24. ***Penicillin***
25. 20-year-old woman presents to the ED with the vaginal itching and discharge. She has a history of chlamydia infection. On exam, there is frothy greenish-yellow vaginal discharge present, but no cervical motion or adnexal tenderness are appreciated. Pregnancy test is negative. The wet mount reveals flagellated protozoa. What is the most appropriate treatment?
26. Clindamycin 300 mg by mouth twice daily for 7 days
27. Fluconazole 150 mg by mouth once
28. ***Metronidazole 2 grams by mouth once***
29. Metronidazole vaginal gel 0.75% daily for 7 days
30. A 38-year-old female presents having found a tick attached to herself after hiking on the East Coast that you identify as an Ixodes species. She is asymptomatic. Prophylaxis is recommended by the Infectious Diseases Society of America if the tick is estimated to have been attached for:
31. Greater than 12 hours
32. Greater than 24 hours
33. ***Greater than 36 hours***
34. Only if symptoms are present regardless of attachment duration
35. A 16-year-old male presents with a non-pruritic rash that has began after hiking in North Carolina. The rash started with the soles of his feet, spread to his ankles, and now involves his chest. What tick special most likely served as a vector?
36. Lone Star Tick
37. ***Dermacentor Andersoni***
38. Ixodes Scapularis
39. Black-Legged Tick
40. A 33-year-old male presents 1 week after hiking and finding a tick attached to himself. He ignored it at the time but now has a rash over his soles, a headache, and muscle aches. What electrolyte abnormality is most likely associated with his condition?
41. ***Hyponatremia***
42. Hypernatremia
43. Hypokalemia
44. Hypocalcemia

**Cumulative Post-Quiz: Learning Objectives 4-6** (infectious rashes, foodborne illnesses, and infectious causes of neuromuscular blockade)

1. A 5yo female is brought in 10 days after having spent the weekend at a lake with his family. When asked about the trip she says “The lake water tasted yummy!” Today he began to have now has profuse, watery diarrhea as well as abdominal cramping and bloating. What is the best treatment?
2. Doxycycline
3. ***Tinidazole***
4. Atovaquone
5. Penicillin G
6. Acyclovir
7. A 16-year-old female presents with 3 days of abdominal pain, vomiting, and bloody diarrhea after having eaten a very rare hamburger a week ago. She now has a hemoglobin of 6 g/dL, platelets of 28/L, a creatinine of 4.5. What treatment is the best choice?
8. Ciprofloxacin
9. Doxycycline
10. ***Supportive Care Only***
11. Trimethoprim/Sulfamethoxazole
12. A 7-year-old male presents with focal right lower quadrant pain, nausea, vomiting, and diarrhea for 1 day. A right lower quadrant ultrasound is negative. What is the classically associated organism?
13. Escherichia coli
14. Rotavirus
15. Shigella
16. ***Yersinia Enterocolitica***
17. A 5-year-old unvaccinated male recently developed fatigue, malaise, and anorexia, followed by a widespread rash associated with pruritus. Wounds appear to be in various stages of healing, some scabbed and other vesicular. What is the most likely diagnosis?
18. Measles
19. Mumps
20. Rubella
21. ***Varicella***
22. A 3-year-old male presents with fevers to 103F, lethargy, and a skin rash. On your exam, he is difficult to arouse, has a delayed capillary refill, and a petechial rash over his trunk and extremities, including palms and soles. What is your next step in management?
23. ***Administer IV ceftriaxone***
24. Administer IVIG
25. Perform lumbar puncture
26. Supportive care only
27. A 32-year-old female with no prenatal care who recently immigrated to the U.S. from Romania delivers at 38 weeks. The child is born with bilateral sensorineural hearing loss, a petechial rash, as well as a patent ductus arteriosus. What test will help you confirm the diagnosis?
28. Cytomegalovirus PCR
29. Herpes simplex virus PCR
30. ***Rubella-specific IgM antibodies***
31. Rapid plasma reagin (RPR)
32. A 54 year old male presents with a complaint of weakness after sustaining a puncture wound to his left calf 10 days ago. Upon arrival, he is afebrile. His wound appears to be healing well with no signs of infection. His neurologic examination demonstrates bilateral ptosis, dysarthria, and upper extremity weakness. What is the most likely cause?
33. Acute flaccid myelitis
34. ***Clostridium botulinum***
35. Clostridium tetani
36. Guillain-Barré syndrome (GBS)
37. A 35-year-old male presents with altered mental status and weakness. On examination, you notice a well healed wound over his arm with multiple puncture marks. He is febrile to 101F and tachycardic to 130 bpm. On exam, he is extremely agitated, with excessive salivation, dysarthria, muscle spasticity, and neck and back hyperextension. What treatment should be initiated?
38. Botulism antitoxin
39. Intravenous Vancomycin
40. ***Supportive care only***
41. Tetanus immune globulin
42. You are describing the benefits of vaccination to a patient’s mother, and she inquires about the clinical features of tetanus. What constellation of symptoms do you describe?
43. Altered mental status, neck and back hyperextension, and hydrophobia
44. Ascending paralysis, respiratory failure
45. Descending paralysis, visual disturbances, and trouble speaking
46. ***Tonic contraction of muscles, inability to open mouth, and neck and back hyperextension***


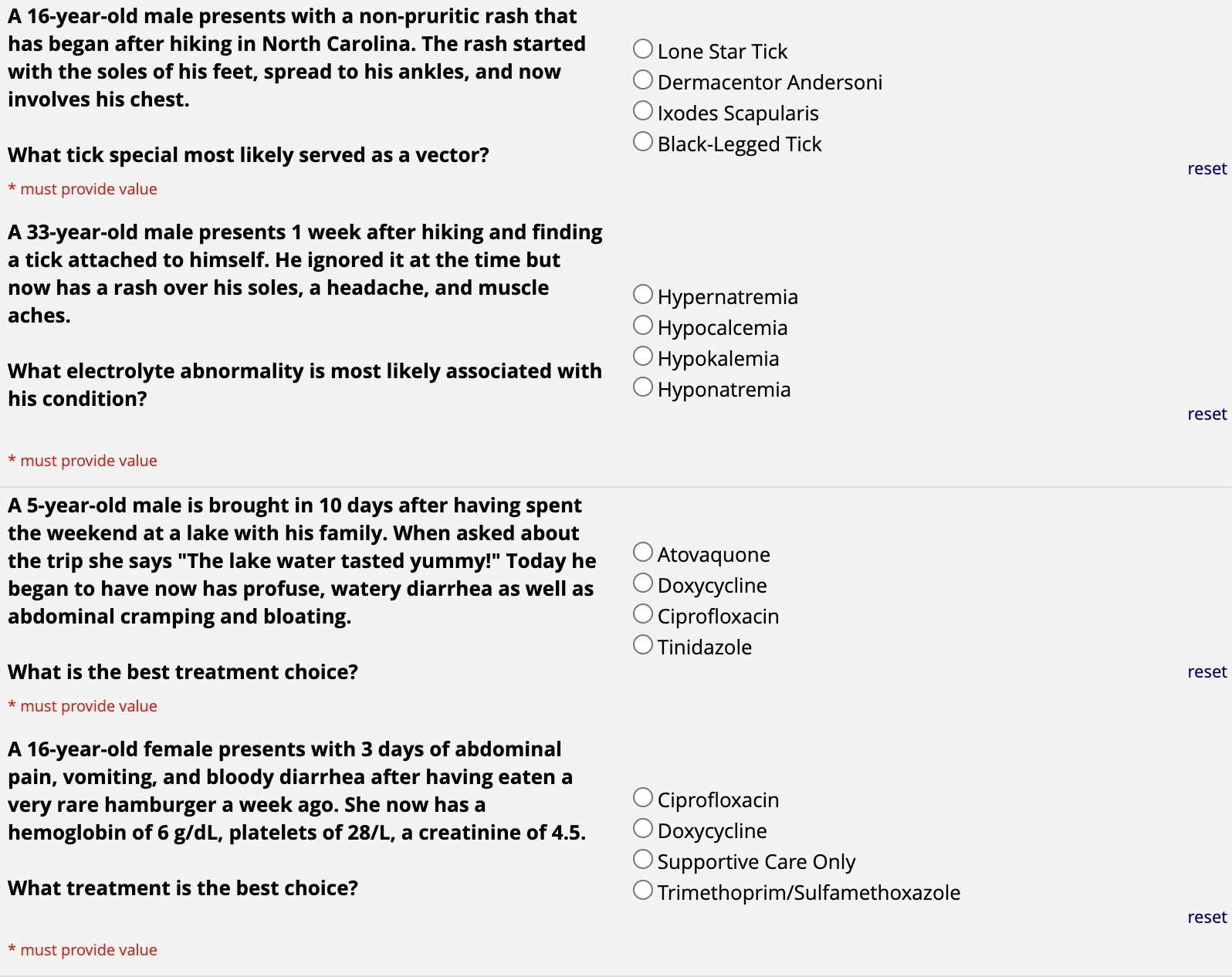


**Intrinsic Motivation Inventory (IMI), Interest and Enjoyment Subscales: Escape Room**

1. I enjoyed the escape room activity very much.
2. Not at all True
3. Somewhat True
4. Very True
5. The escape room activity was fun to do.
6. Not at all True
7. Somewhat True
8. Very True
9. I thought the escape room was a boring activity.
10. Not at all True
11. Somewhat True
12. Very True
13. The escape room activity did not hold my attention at all.
14. Not at all True
15. Somewhat True
16. Very True
17. I would describe the escape room activity as very interesting.
18. Not at all True
19. Somewhat True
20. Very True
21. I thought the escape room was quite enjoyable.
22. Not at all True
23. Somewhat True
24. Very True
25. While I was doing the escape room activity, I was thinking about how much I enjoyed it.
26. Not at all True
27. Somewhat True
28. Very True

**Intrinsic Motivation Inventory (IMI), Interest and Enjoyment Sub-scales: Didactic Lecture**

*(where 0= not at all true, 4= somewhat true, 7=very true as represented by slider bar)*

1. I enjoyed the didactic lecture very much.
2. Not at all True
3. Somewhat True
4. Very True
5. The didactic lecture was fun to watch.
6. Not at all True
7. Somewhat True
8. Very True
9. I thought the didactic lecture was boring.
10. Not at all True
11. Somewhat True
12. Very True
13. The didactic lecture did not hold my attention at all.
14. Not at all True
15. Somewhat True
16. Very True
17. I would describe the didactic lecture as very interesting.
18. Not at all True
19. Somewhat True
20. Very True
21. I thought the didactic lecture was quite enjoyable.
22. Not at all True
23. Somewhat True
24. Very True
25. While I was doing the didactic lecture, I was thinking about how much I enjoyed it.
26. Not at all True
27. Somewhat True
28. Very True
